# “Integración de los equipos de salud y la comunidad”: Discurso pronunciado en la Universidad Nacional y Popular de Buenos Aires

**DOI:** 10.18294/sc.2025.5764

**Published:** 2025-07-03

**Authors:** Mario Testa

**Affiliations:** 1 (1925-2024). Médico. Doctor Honoris Causa, Universidad Federal de Bahía, Brasil. Doctor Honoris Causa, Universidad Nacional de Lanús, Argentina. Ex profesor, Instituto de Salud Colectiva, Universidad Nacional de Lanús, Buenos Aires, Argentina. Universidad Nacional de Lanús Instituto de Salud Colectiva Universidad Nacional de Lanús Buenos Aires Argentina; Universidad Federal de Bahía Brasil

**Keywords:** Educación Médica, Proceso de Enseñanza-Aprendizaje, Universidades, Argentina, Medical Education, Teaching-Learning Process, Universities, Argentina

## Abstract

En este discurso, pronunciado en la clase inaugural del curso de trabajo premédico de la Facultad de Medicina de la Universidad Nacional y Popular de Buenos Aires, en 1973, Mario Testa plantea una profunda crítica al modelo educativo heredado del proyecto oligárquico liberal, caracterizado por el individualismo, el facilismo y la desvinculación con la realidad del país. Propone una transformación integral de la universidad, orientada a romper su estructura feudal y a construir una institución comprometida con las necesidades sociales y políticas de la nación. Frente al tradicional modelo médico basado en el prestigio, el poder y la competencia, Testa impulsa una nueva concepción de la medicina como práctica colectiva y solidaria, inscrita en un proyecto nacional y popular. Destaca la necesidad de formar equipos de salud integrados con la comunidad, en los que médicas y médicos dejen de ocupar un lugar central para convertirse en un trabajador más dentro de un proceso colectivo centrado en la resolución de los problemas del pueblo. Por último, postula que la Facultad de Medicina, transformada en Facultad de Ciencias de la Salud, debe regirse por la medicina social, para formar profesionales comprometidos con el trabajo en equipo, la crítica y la participación activa en la construcción de un país más justo.

## Abstract (en)

In this speech, delivered at the inaugural class of the pre-medical course at the Faculty of Medicine of the Universidad Nacional y Popular de Buenos Aires, in 1973, Mario Testa presents a profound critique of the educational model inherited from the liberal oligarchic project, marked by individualism, superficiality, and detachment from the country’s reality. He calls for a comprehensive transformation of the university, aimed at dismantling its feudal structure and building an institution committed to the social and political needs of the nation. Confronting the traditional medical model grounded in prestige, power, and competition, Testa advocates for a new conception of medicine as a collective and solidarity-based practice, embedded in a national and popular project. He emphasizes the need to form health teams integrated with the community, where physicians no longer occupy a central role but instead become one more worker in a collective process focused on solving the people’s problems. Finally, he asserts that the Faculty of Medicine, transformed into a Faculty of Health Sciences, must be guided by social medicine, in order to train professionals committed to teamwork, critical thinking, and active participation in the construction of a more just country.

## INTRODUCCIÓN

El texto que reproducimos corresponde a uno de los tantos discursos que Mario Testa pronunció en su recorrido como delegado interventor y decano de la Facultad de Medicina de la Universidad Nacional y Popular de Buenos Aires, entre 1973 y 1974. En este caso, se trata del discurso pronunciado en la clase inaugural del curso de trabajo premédico de la Facultad de Medicina, en el marco de un ambicioso proceso de transformación institucional orientado a vincular la formación médica con las necesidades reales del país, desde una perspectiva nacional, popular y solidaria.

Mario Testa (1925-2024) fue un médico sanitarista, docente e intelectual argentino, ampliamente reconocido por su aporte al pensamiento crítico en salud colectiva en América Latina. Su obra, marcada por una fuerte articulación entre ciencia, política y ética, contribuyó a consolidar una mirada crítica sobre el campo de la salud, centrada en la construcción de equipos y la participación comunitaria. Publicó numerosos artículos científicos, entre ellos diversos artículos en la revista *Salud Colectiva*^1^^,^^2^^,^^3^^,^^4^^,^^5^, y es autor de obras fundamentales como *Pensar en salud*^6^, *Saber en salud: La construcción del conocimiento*^7^, *Pensamiento estratégico y lógica de programación: El caso salud*^8^ y *Medicina del trabajo al servicio de los trabajadores*^9^, publicadas en la colección Cuadernos del ISCo.

Este discurso se inscribe en un momento clave del recorrido intelectual y político de Mario Testa, al que él mismo haría alusión años más tarde en la entrevista publicada en el libro *Memoria de planificadores…* de Dora Barrancos y Eugenio Vilça Mendez: “...Es cierto, antes hacía ciencia, no hacía política, después hice política, no hice ciencia... Pero fue a partir de 1976, el momento de la derrota, cuando se genera el espacio necesario para repensar esta articulación ciencia-política”^10^. 

El texto que aquí se presenta forma parte de ese “después hice política”, una etapa marcada por el intento de transformar las instituciones universitarias desde adentro, al calor del retorno de Juan Domingo Perón al gobierno y de los debates que atravesaban a la medicina, la salud y la universidad en la Argentina de la década de 1970.

## DISCURSO PRONUNCIADO EN LA CLASE INAUGURAL DE LA CARRERA DE MEDICINA

Este curso se inicia hoy de una manera muy auspiciosa porque produce una profunda alegría ver el aula magna, tan cargada de pesados acontecimientos académicos, colmada de una juventud, que es la única motivación de nuestro trabajo. Pero a esta juventud nosotros venimos a plantearles duras exigencias, exigencias que no escatimamos para nosotros mismos, que estamos trabajando todo lo que podemos, dando todo lo que podemos dar, para que ustedes se conviertan, de una esperanza que son ahora, en la realidad futura de nuestro país. Pero esas exigencias que venimos a plantearles escapan del terreno puramente personal, individual de cada uno de ustedes y de cada uno de nosotros. Cuando digo ustedes y nosotros estoy haciendo una aparente separación en la cual ustedes son los que se sientan de aquel lado y nosotros en un estrado que me molesta que sea un poco más alto, porque da una apariencia de que estamos un poco por encima. Nosotros no lo interpretamos así, intentamos y entendemos que tenemos algo que construir todos juntos y que tenemos que hacerlo a partir de algo así como una tierra arrasada que nos dejaron estos últimos 18 años, años que queremos empezar a olvidar para reconstruir lo que fuimos alguna vez y para plantearnos una tarea de futuro.

Quiero iniciar esto planteando el problema del tránsito que ustedes deben hacer en este momento, de pasar de ser lo que sin duda todavía son: estudiantes secundarios, a ser estudiantes universitarios. Este tránsito no es fácil, podemos decirlo por experiencia. No es fácil en este momento porque ustedes vienen de una escuela que estaba inserta en el proyecto de la oligarquía liberal, y a pesar de las resistencias que ese proyecto puede haber gestado en ustedes mismos, seguramente dejó su marca en ustedes tal como la dejó en nosotros también.

**Figura 1:**
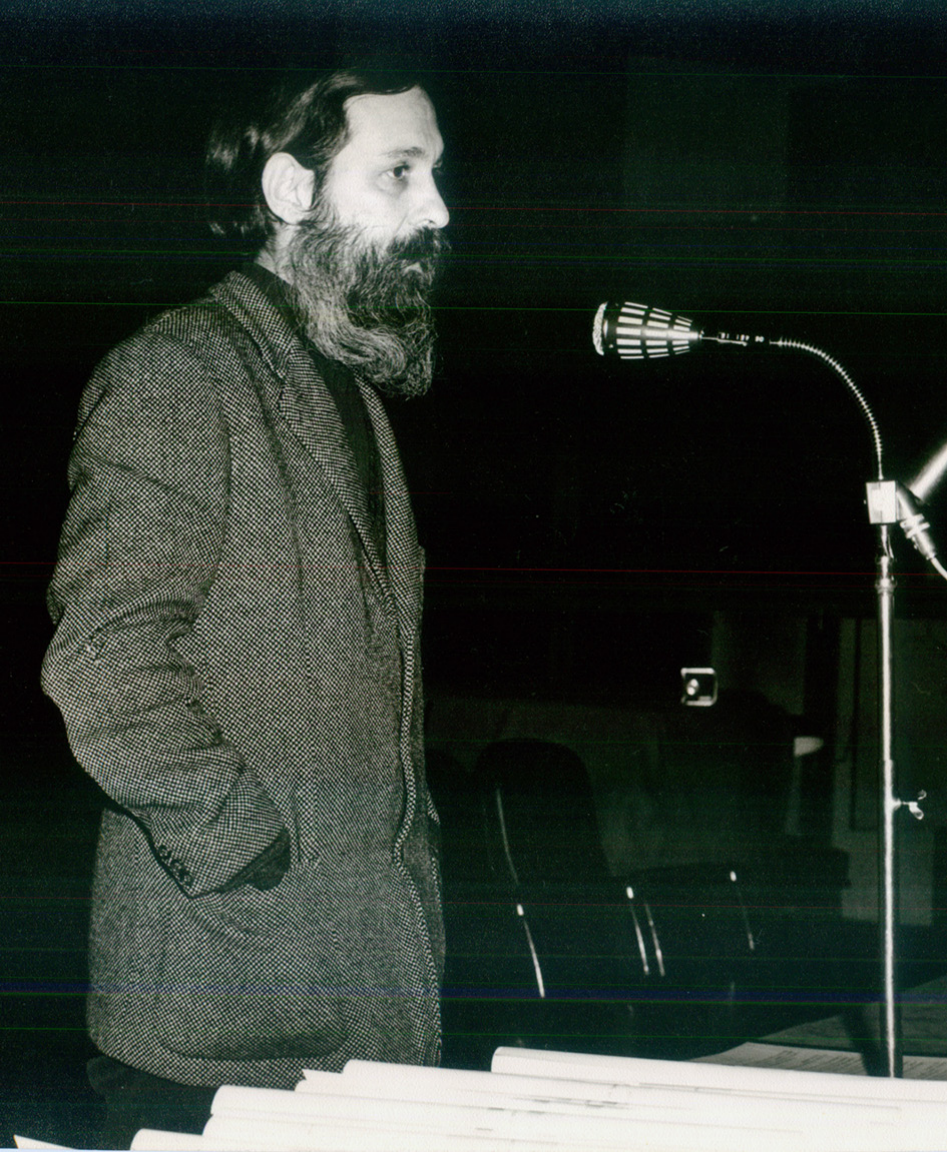
Mario Testa (1925-2024) en los años de delegado interventor y decano de la Facultad de Medicina de la Universidad Nacional y Popular de Buenos Aires, entre 1973 y 1974.

Fuente: Fondo Mario Testa, Centro de Documentación Pensar en Salud, Instituto de Salud Colectiva, Universidad Nacional de Lanús.

## CARACTERÍSTICAS DEL ESTUDIANTE SECUNDARIO

Quiero comenzar por analizar brevemente cuáles son las características del estudiante secundario que intenta llegar a la universidad. Es un estudiante que, por las condiciones mismas que ese proyecto liberal gestó, proviene de una cierta extracción social; tiene un contenido de clase, porque no toda la población, no todos los adolescentes pueden llegar todavía a la escuela secundaria, menos podían en el momento en que ustedes transitaron por las aulas de la escuela secundaria. Eso se puede mostrar con cifras. Se puede ver a través de analizar la pirámide demográfica escolar que, partiendo de los años iniciales de la escuela primaria, se va afilando progresivamente, y se va afilando no porque se pongan vallas para una determinada clase social, sino porque la situación misma de la clase más pobre, sus condiciones de existencia, son las causantes de que muchos de los que ingresan en la escuela primaria no la terminen, y de que los que la terminan, en muchos casos, no puedan ingresar a la escuela secundaria o tengan que abandonarla a poco andar. Eso va creando en la escuela secundaria una cierta composición de clases en la cual seguramente muchos de ustedes, la mayoría posiblemente, están o se los podría categorizar. Esa extracción social genera, necesariamente, ciertas actitudes que son coherentes con lo que el proyecto oligárquico liberal necesita para su perpetuación. Las actitudes a las que hago referencia son actitudes individualistas, que son las que muchos, de ustedes traen aquí.

La escuela secundaria también genera otras cosas, por un lado, a través de su desvinculación con el sistema productivo, y, por otro lado, de la utilización de un sistema represivo. La desvinculación con el sistema productivo no es característica de la escuela secundaria, ya que existe también para la mayoría de los estudiantes universitarios. El sistema represivo tiende a que el estudiante “se porte bien” aunque no aprenda. Estos dos elementos generan una forma de estudio que fue y sigue siendo hasta ahora, característica de los estudios en la escuela media y que podría definirse como el “facilismo”. El facilismo se debe a que los estudiantes no están en contacto con la actividad más importante que se desarrolla en este país o en cualquier otro, que es el trabajo. Los estudiantes no están acostumbrados a trabajar.

Los dos aspectos que hemos señalado, la actitud individualista y la forma de estudio facilista, condicionan las características que traen desde su formación previa los estudiantes que nosotros recibimos.

## ROLES DEL ESTUDIANTE SECUNDARIO

Pero hay más, deberemos ver también cual es el rol que el estudiante juega en la escuela secundaria frente al contexto en el que se ve envuelto; contexto que está formado por sus propios compañeros, por sus profesores, por los trabajadores en general, y por la familia de la que provienen.

Lo que podemos observar en la escuela secundaria en este momento, y más aún en los años pasados, es una actitud de competencia frente a los compañeros: hay que ser mejor alumno, hay que sacar mejores notas, hay que destacarse frente a los demás porque a eso se ve estimulado el estudiante por la formación que se le imparte. Frente a los profesores existe una actitud que generalmente es de subordinación, porque el profesor es el que sabe o el que manda y, como eso es incuestionable, el estudiante se subordina sin tener alternativas o, a veces, con algunas manifestaciones de rebeldía, pero sin mayores consecuencias. 

Con respecto a los trabajadores del país, no existe prácticamente ninguna relación. Son dos mundos aparte. Parecería que los estudiantes no tienen nada que ver con lo que se hace en el país. Frente a la familia, la relación reproduce un poco la que hay con los profesores, puede ser de subordinación o de enfrentamiento; pero un enfrentamiento que no significa otra cosa que una subordinación disimulada.

## MOTIVACIÓN DEL ESTUDIANTE DE MEDICINA

Frente a las características y roles del estudiante secundario, corresponde analizar cuáles son las motivaciones de los estudiantes que quieren ingresar a la Facultad de Medicina. Estas motivaciones se sintetizan en: prestigio, poder, dinero y servicio.

La mejor ejemplificación del prestigio de la profesión médica se encuentra tal vez en un libro que no es citado frecuentemente entre la bibliografía académica. Permítanme citar aquí una historia de “Mafalda”, en la cual aparece el padre de Mafalda, que es un empleado de un banco, que está en una playa donde se encuentra con un señor, los dos en pantalón de baño, sin ningún signo evidente de estatus, y entonces este señor le pregunta al padre de Mafalda qué hace y recibe como respuesta: empleado de banco. Ante la devolución de la pregunta, el otro contesta: “yo soy doctor”. La imagen gráfica reproduce la imagen mental que ambos se hacen de la situación: el padre de Mafalda aparece empequeñecido y avergonzado y el otro señor aparece muy derecho, sacando pecho, agrandado y parado sobre un pedestal. Esta es la imagen que generalmente tenemos de los médicos; imagen que los médicos hemos construido cuidadosamente, y que ha sido aceptada como tal por la generalidad de la población.

El poder es otra de las motivaciones que nos pueden llevar a estudiar medicina. Para ejemplificar lo que es el poder del médico, recordemos que el médico es aquel personaje delante del cual la gente se desnuda. Creo que no existe una manifestación más real del poder que esa, la existencia de alguien ante quien todos eliminamos los pudores y nos desnudamos físicamente, que es como si nos desnudáramos el alma.

El dinero, por supuesto, es otra de las motivaciones que nos pueden llevar a estudiar esta carrera. La idea de que el médico es un personaje que gana mucho dinero es un mito cuya irrealidad vamos a tener que mostrar, analizando la realidad sanitaria nacional. Si en algún pasado ese mito fue una realidad, ello se ha perdido definitivamente para la mayoría de los médicos. Hoy el médico no es un personaje que se pueda enriquecer fácilmente, como era habitual años atrás, cuando el país no había sufrido todavía lo que significó el peronismo con su ascenso de las grandes masas al poder y que ahora vuelve a comenzar con este nuevo gobierno de Perón.

No todas las vocaciones son como las que hemos citado hasta aquí. Hay también los que vienen a la Facultad de Medicina por una vocación de servicio. Servir a los demás, servir al pueblo es una de las motivaciones más nobles que cualquiera pueda tener. Pero también es bueno señalar que esta misma vocación de servicio muchas veces está vista desde una perspectiva individual, en la cual el principal personaje sigue siendo el médico, el médico que sirve a los demás.

## UBICACIÓN DE LA UNIVERSIDAD

Frente a lo descrito hasta aquí, que llamaría el contexto del lado de los estudiantes, quiero plantear cuál es la ubicación de la universidad, y de la facultad en particular. En términos tradicionales esta ubicación se podría caracterizar como una situación feudal, en la que la universidad se mantiene como un feudo frente al país, la facultad como un feudo frente a la universidad y cada cátedra como un feudo frente a la facultad.

No es nueva la idea de la universidad como feudo. Esto ocurrió prácticamente desde que la universidad fue creada y se mantiene hasta hoy, unas veces como “isla democrática”, otras como intento de “isla socialista”, pero siempre como isla, como cosa aparte de lo que es el país real, alejada de sus problemas. Esta situación se dio independientemente de las buenas o malas intenciones de quienes la gestaron. El quehacer universitario nunca se volcó de manera efectiva sobre los problemas nacionales.

No solo la universidad mantuvo esta situación con respecto al país, sino que cada una de las facultades que la componen se transformó en un feudo respecto de la universidad, lo cual significó la desconexión con respecto a lo que es el quehacer científico y la enseñanza en un sentido global, ya que la ciencia no es una cosa compartimentada que se pueda separar en pedazos aislados. No existe una parte de la ciencia que corresponda a una facultad u otra, no hay límites entre el conocimiento. Sin embargo, esos límites se establecieron artificialmente para formar los feudos que se transformaron en nuestras facultades, de manera tal que la actual Universidad Nacional y Popular de Buenos Aires no es más que una confederación de facultades islas. Creemos que esto es un tremendo error.

Pero hay más. Dentro mismo de nuestra Facultad, las cátedras son feudos con respecto a la misma Facultad. Esto crea situaciones de arbitrariedad, muchas veces de prepotencia entre la conducción de la Facultad y de las cátedras. En las cátedras mismas se origina un alejamiento notable de lo que son los problemas del país y las necesidades de los alumnos. Esta situación, se ve reforzada tremendamente por los procedimientos mediante los cuales se recluta el personal docente, en especial, a los profesores. Esos procedimientos tienden a perpetuar el dominio de la universidad por esos grupos que son sus tradicionales “dueños”. Esta es la universidad en situación feudal y esa es la situación tradicional.

En la actualidad existe un intento de romper esa situación que se da en la universidad a través de los cambios estructurales que han sido propuestos por la actual intervención, que han sido conducidos -con clara mentalidad y definido propósito de romper esa situación feudal- por el compañero a quien en este momento rindo mi homenaje, que es quien ha guiado la intervención en la universidad desde mayo hasta hoy: el compañero Rodolfo Puiggrós.

Puiggrós, que es un hombre de una clara mentalidad nacional y popular, avalada por una profusa obra escrita, conoce a fondo este problema. Es bajo su conducción, repito, que se ha gestado un proyecto que tiende a eliminar la separación de la universidad con respecto al país y de las facultades con respecto a la universidad. Es posible, o mejor dicho es seguro, que ustedes participarán en la discusión de las modificaciones propuestas. La ocasión para ello será cuando la propuesta sea sometida a discusión por la base estudiantil, de la cual desde hoy ustedes forman parte. Esa discusión también se hará en el ámbito de docentes y trabajadores universitarios, para luego ampliarse a todo el país, para que todos tengamos la oportunidad de analizar, discutir y aprobar si estamos de acuerdo, y creemos que esas transformaciones van a significar la ubicación definitiva de la universidad dentro de lo que es el conjunto del país y que van a comenzar a eliminar las diferencias, o las separaciones y muchas veces las duplicaciones que existen entre nuestras distintas casas de estudio.

En esta Facultad estamos tratando también de implementar medidas que signifiquen romper esa situación feudal. Hemos estado tratando de introducir cambios en los planes de estudio que rompan el aislamiento que señalaba antes. Esos cambios también van a ser propuestos para la discusión amplia de todos los que tienen que ver con este quehacer universitario. En ese sentido, es muy posible que los estudios que ustedes inician hoy, porque ya los consideramos a ustedes estudiantes universitarios desde el momento que inician este curso, tengan características muy diferentes a los estudios que nosotros en cierta forma padecimos, y aún distintos a los que en este momento se están cursando en esta Facultad. La tarea es ímproba porque hemos tenido muy poco tiempo para gestar este proyecto de cambio curricular, además de conducir esta casa de estudios en tiempos difíciles.

A nivel de las cátedras, que señalábamos como otro de los elementos en los que existe una situación feudal, también se están tratando de implementar cambios que quiebren esa situación, y los cambios los estamos tratando de realizar revisando el reclutamiento de los docentes y a través del cambio de los procedimientos pedagógicos. Tenemos que aprender a aprender y tenemos que aprender a enseñar.

El propósito general de todos estos cambios que estamos proponiendo no es otro que el de transformar a nuestra Facultad de Medicina en una fábrica del producto que el país reclama. Queremos que la Facultad se ponga a trabajar para el país, no para que forme profesionales individualistas cuyo objetivo es “ganarse la vida”.

**Figura 2:**
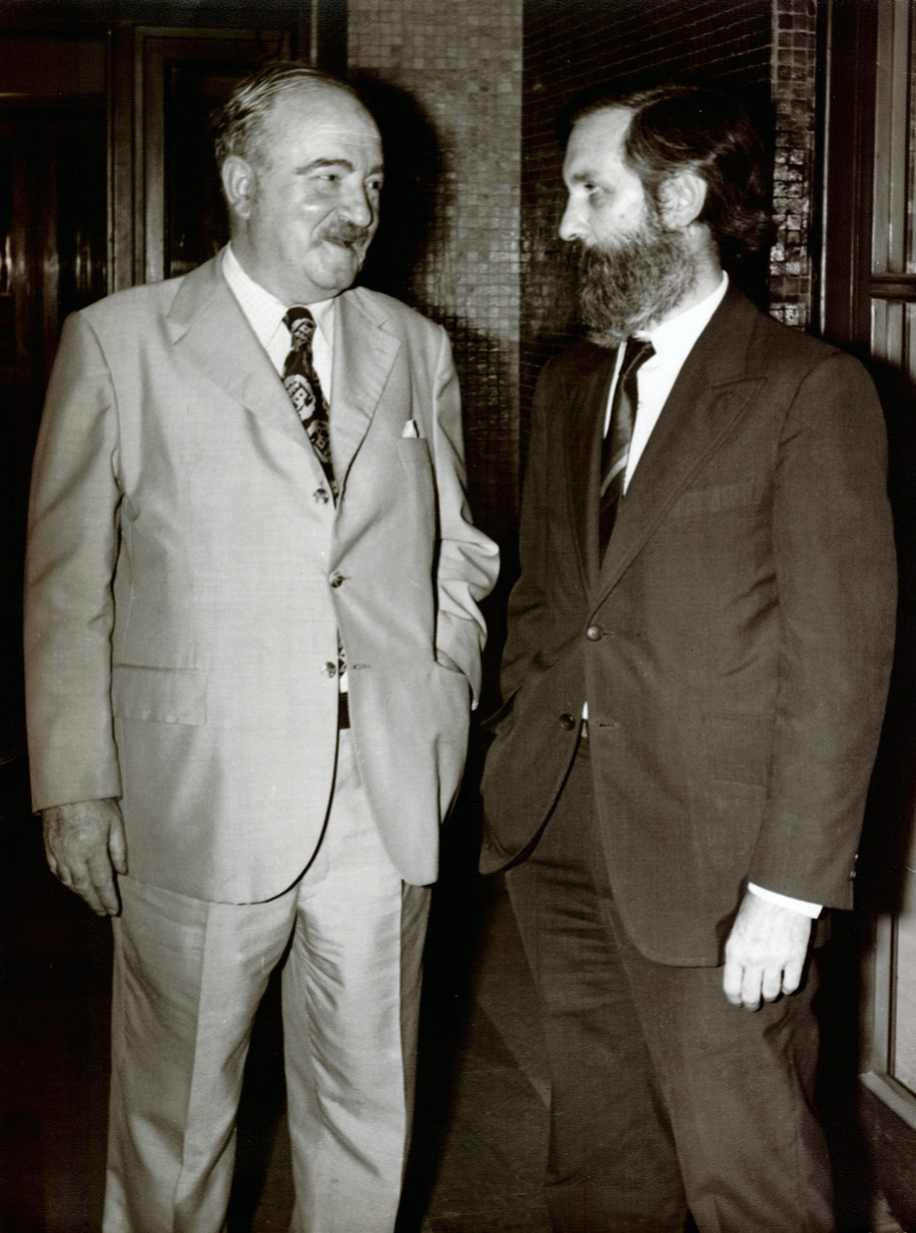
Rodolfo Puiggrós (1906-1980) y Mario Testa (1925-2024).

Fuente: Fondo Mario Testa, Centro de Documentación Pensar en Salud, Instituto de Salud Colectiva, Universidad Nacional de Lanús. Nota: Foto tomada entre 1973 y 1974, momento en que Mario Testa era delegado interventor y decano de la Facultad de Medicina de la Universidad Nacional y Popular de Buenos Aires, y Rodolfo Puiggrós era rector de la misma institución.

**Figura 3:**
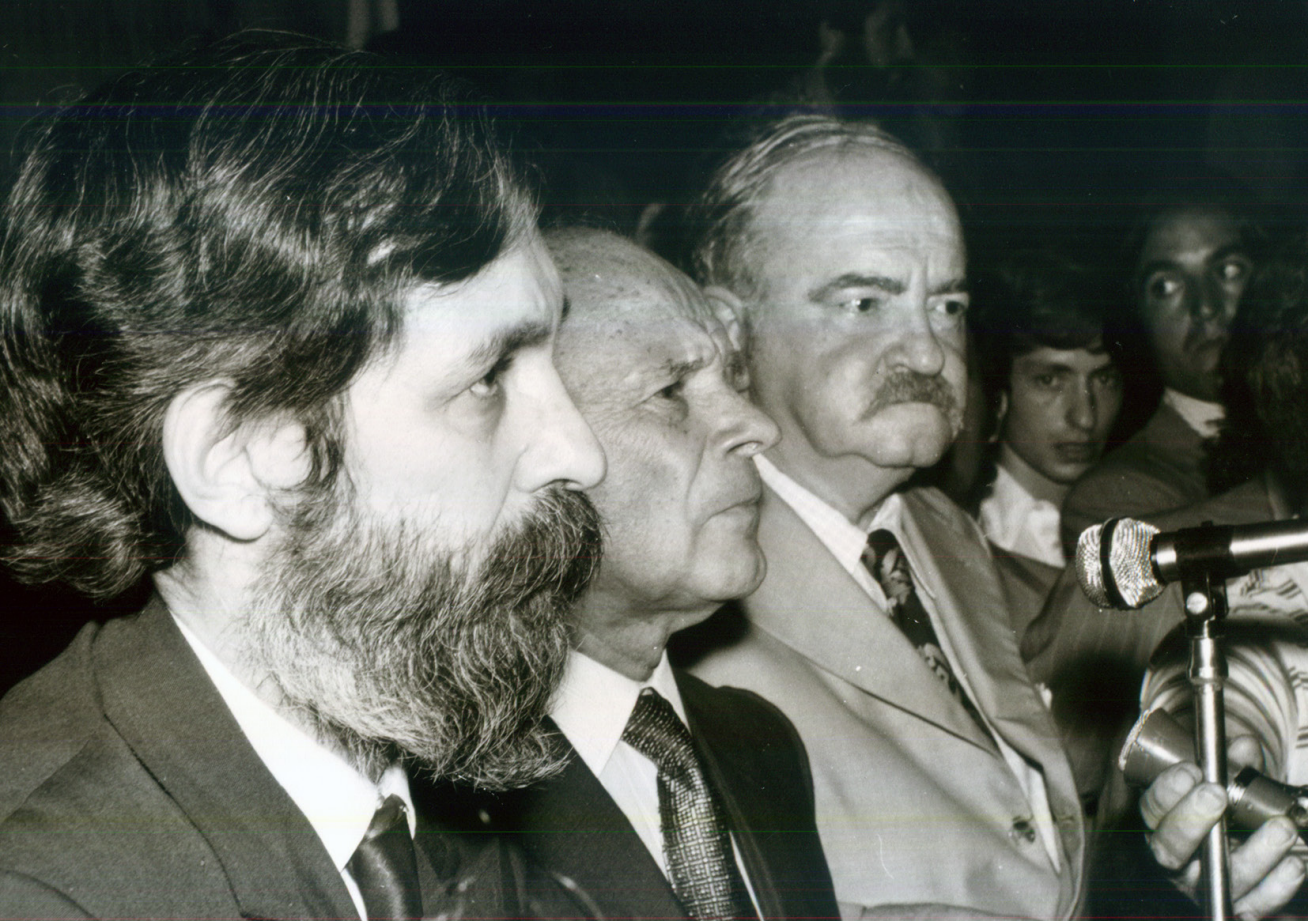
Mario Testa (1925-2024), Vicente Solano Lima (1901-1984) y Rodolfo Puiggrós (1906-1980).

Fuente: Fondo Mario Testa, Centro de Documentación Pensar en Salud, Instituto de Salud Colectiva, Universidad Nacional de Lanús. Nota: Foto tomada entre 1973 y 1974, momento en que Mario Testa era delegado interventor y decano de la Facultad de Medicina de la Universidad Nacional y Popular de Buenos Aires, y Rodolfo Puiggrós era rector de la misma institución. En medio de ambos se encuentra Vicente Solano Lima, por entonces vicepresedente de la Nación.

## EL PAÍS LIBERADO

Queremos que la Facultad trabaje para el país. Esto nos lleva a analizar cuál es el país a que ustedes van a tener que ofrecer sus servicios, el país al que se van a enfrentar cuando salgan de la facultad a la que entran hoy.

Ese país se define por un proyecto político que es totalmente distinto a ese otro proyecto del cual venimos y que nosotros definimos como la oligarquía liberal. Este nuevo país se define con siete millones y medio de votos a favor del general Perón. Hay dos frases que están entre las que se conoce como las 20 verdades del justicialismo que hacen referencia a lo que ese país va a ser. Todas las 20 verdades hacen referencia al país, pero deseo rescatar aquí a dos de ellas: “el gobierno hará lo que el pueblo quiera” y “este país es una comunidad organizada y un pueblo libre”. Esto hace mención a dos problemas. El gobierno hará lo que el pueblo quiera significa participación, participación de todos, participación del pueblo. Lo que se intenta entonces, a través del gobierno peronista, es crear una sociedad participante. Somos una comunidad organizada significa fundamentalmente solidaridad. Lo que el peronismo quiere construir como país es un pueblo solidario. Lo cual nos lleva a pensar a nosotros cómo debemos interpretar esto, o sea, cómo va a ser la medicina en el proyecto político peronista.

Está claro que, en el provecto oligárquico liberal, la medicina era individualista y comercializada, se definía por el mercado existente entre el médico y el enfermo y de estos el personaje más importante, obvio es decirlo, era el médico, no el enfermo.

En el proyecto peronista se está gestando otra medicina que es totalmente distinta a la que se define de esa manera entre médico y enfermo. En esta nueva medicina la relación se establece entre el equipo y la salud. El equipo, en esta concepción, no es solamente el médico, sino que es todo un complejo de fuerzas de trabajadores que son los que enfrentan un problema que la comunidad tiene. Y esta diferencia es fundamental. Nosotros entendemos que el médico debe dejar de ser el personaje principal, ese personaje de Mafalda, para pasar a ser ahora un miembro más de un equipo de trabajadores que tiene, junto con la población y no con un paciente individual, un problema que resolver.

Este cambio de enfoque hace que nosotros tengamos que adaptarnos ante esta situación y revalorar lo que tenemos que enseñar, cómo tenemos qué hacerlo y cómo tenemos que discutirlo todos juntos, para que juntos podamos resolverlo. Y aquí viene nuestra primera exigencia, y la fundamental, hacia los estudiantes que empiezan a acompañarnos en esta tarea. Nosotros les exigimos a ustedes una participación plena, decidida y entusiasta con respecto a lo que tenemos que realizar. Exigimos la crítica de lo que hacemos, exigimos que nos hagan propuestas y la discusión amplia de todos los problemas que surjan en el futuro. Esta es una exigencia nuestra a todos los estudiantes de esta Facultad.

## EL CURSO DE TRABAJO PREMÉDICO

Corresponde ahora ver cuál es el papel que asignamos a este curso de trabajo premédico. Nuestro ideal es que este curso no exista. En consecuencia, entendemos que lo que iniciamos hoy es algo que debe ser radicalmente transformado en el futuro.

Estamos pensando desde la universidad, pero no solamente para la universidad. En una escala más amplia estamos pensando en un proyecto educativo que no puede limitarse al ámbito universitario, que tiene que afectar a todos los niveles de la enseñanza para que ese estudiante secundario que caractericé, y que tal vez caricaturicé un poco al principio, pueda llegar a la universidad sin las actitudes con que en este momento llegan nuestros estudiantes. En consecuencia, nuestro proyecto implica la transformación de lo que es la escuela media. La escuela media no puede seguir siendo un gestador de actitudes individualistas y de formas de estudio antipedagógicas. Cuando los estudiantes lleguen a la universidad con las actitudes no individualistas y no facilistas con que llegan en este momento, y cuando lleguen habiendo aprendido lo que es el país real y estando insertos en un sistema productivo, este curso habrá dejado de tener razón de ser. Nosotros esperamos que en muy breve plazo eso pueda ocurrir, claro que el breve plazo se define, en este caso, en términos de algunos años.

En los momentos actuales, el curso intenta lograr que el estudiante que llega perciba sus características reales, se dé una imagen real de en qué proyecto se está metiendo y que tenga además una actitud crítica frente a sí mismo y frente a lo que nosotros queremos que haga.

Este curso no es un refuerzo de lo no aprendido en la escuela media -la Biología, la Química, la Matemática- porque esas materias que tienen que saber específicamente para ser médicos o para actuar dentro del equipo de salud -hablando con una terminología más adecuada con nuestro proyecto- eso lo van a aprender en la asignatura que corresponda, que tendrán que cursar según los planes de estudio que se gesten a partir de ahora. Antes había que estudiar esas materias en los cursos de ingreso, como una forma de poner una valla para ingresar a la universidad o en esta facultad. Ahora esas materias no se van a estudiar en este curso, que no llamamos de ingreso, sino de trabajo premédico, porque esas materias van a ser estudiadas en la medida que sea necesario, dentro de lo que podríamos llamar el curriculum habitual de la carrera que se siga.

Este curso tampoco es un “meloneo” político para peronizar a los estudiantes. No tenemos la intención de lavar el cerebro a nadie. Los estudiantes que ingresen aquí, y que trabajen con la concepción de que hay que trabajar para el país, se van a convencer por sí mismos de que deben apoyar el proyecto político que se ha gestado en el país, a través de oír lo que el país haga, y no de otra manera. De modo que nosotros no intentamos, a través de este curso, y quiero que esto quede bien claro, peronizar a los estudiantes. Sí queremos que adquieran una profunda conciencia crítica que creemos que la escuela secundaria no ha ayudado a formar. Por eso es que el curso sí intenta ubicar al estudiante en la realidad argentina de 1973, sí intenta deshacer los vicios que el estudiante arrastra de un pasado que estaba en desacuerdo con la actitud que se necesita para enfrentar con éxito los estudios que inicia, y sí intenta darle algunos elementos para que evalúe sus actitudes personales y sociales en este episodio que hoy se inicia y que es crítico para su futuro y el del país.

## PAPEL DE LA FACULTAD DE MEDICINA

Quiero evaluar brevemente el papel de la Facultad de Medicina frente a este panorama que he esquematizado rápidamente. La Facultad de Medicina actual entiende que lo que es necesario formar no son individuos con conocimientos particularizados que los van a llevar a una situación muy posiblemente frustrante. Lo que la Facultad de Medicina actual intentará hacer es formar gente con mentalidad de equipo.

El equipo de salud no es simplemente el médico con algunos ayudantes que andan a su alrededor facilitándole el trabajo; el médico, en el equipo de salud, es un trabajador más. El equipo de salud es el que se encarga entonces de acercarse a la comunidad para resolver un problema en conjunto, y no en conjunto solamente con el equipo de salud, sino con la concepción de que debe integrarse a la comunidad para resolver, con ella, los problemas que esa comunidad tiene. Junto con la comunidad, no separado de ella, no enfrentado a ella, no dominándola, sino participando con ella en la resolución de los problemas. Dentro de esta concepción, el médico tradicional, prestigioso, poderoso, que conocíamos en el pasado, se acabó. Esto ya no puede seguir siendo así, porque el país lo exige. Ese país, que no entendió este problema, o no lo quiso entender, a través de las conducciones que padeció durante estos años pasados, distorsionó por completo esta situación.

En este momento, padecemos de terribles carencias en el personal de salud, que no son precisamente de médicos. Son de muchos otros de los componentes del equipo de salud. Esto se debe, fundamentalmente, a la falta de una concepción de trabajo en equipo y es importantísimo que nuestros estudiantes lo entiendan. Porque nosotros queremos transformar esta Facultad, de una Facultad de Medicina en una Facultad de Ciencias de la Salud, una Facultad que geste esos equipos que el proyecto político peronista o, mejor dicho, el proyecto político de la Nación que ahora se pone en marcha quiere ofrecer al país.

La orientación que queremos es entonces la que tienda a resolver los problemas del pueblo, lo cual plantea a nuestros estudiantes una nueva exigencia, que es el desplazamiento de una mentalidad y una actitud individualista hacia una mentalidad y una actitud social o colectiva.

En este sentido, nosotros ya hemos comenzado a actuar a través del cambio en los planes de estudio que les comentaba hace un rato. Esos cambios incluyen algunos aspectos fundamentales tales como la formación coordinada de todo el equipo de salud, para que desde el inicio de la carrera se empiece a pensar en esos términos. Pero no son esos los únicos cambios que estamos tratando de instrumentar. Queremos, además, dar a la carrera de Medicina, o mejor de Ciencias de la Salud para llamarla con el nombre que vamos a utilizar desde ahora, un contenido de medicina social. La medicina social siempre existió en la Facultad de Medicina, pero con características de algo secundario, como una cátedra con un programa que había que sacarse de encima de la forma más rápida posible porque era un inconveniente, una molestia más, para llegar a lo importante que era el médico individualista.

**Figura 4:**
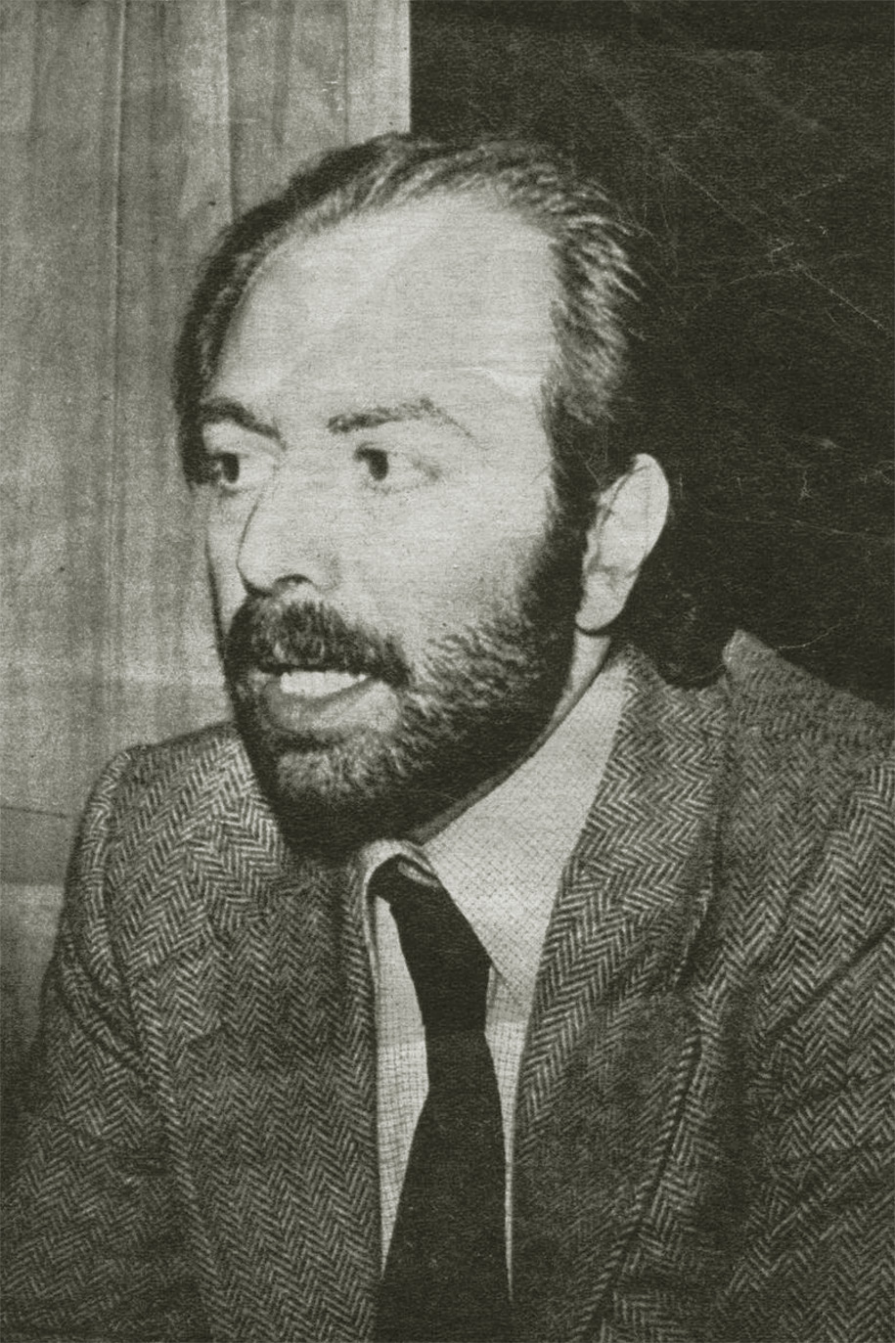
Ricardo Saiegh, primer director del Instituto de Medicina del Trabajo.

Fuente: Fondo Mario Testa, Centro de Documentación Pensar en Salud, Instituto de Salud Colectiva, Universidad Nacional de Lanús. Nota: El Instituto de Medicina del Trabajo fue creado en la Facultad de Medicina pocos meses después de la intervención de la Universidad de Buenos Aires (UBA), en julio de 1973^11^.

Entendemos que el enfoque de la medicina social debe regir desde este momento la concepción de la Facultad, de sus docentes y de sus estudiantes, porque es lo que el país nos reclama. En ese sentido es que estamos trabajando para producir esa transformación tan necesaria.

Dentro de la misma línea de pensamiento es que hemos creado el Instituto de Medicina del Trabajo, por entender que es el trabajo la instancia superior de la actividad social del país. A través de la labor que en tan breve lapso ha desarrollado este Instituto hemos comenzado a percibir con más claridad cuáles son los graves problemas de salud que una forma alienada de trabajo produce en los trabajadores. Un solo dato para expresarlo sintéticamente: en Argentina no existe un solo minero jubilado. Esto es algo que no aparece en los periódicos, pero que se puede detectar fácilmente cuando uno se pone a examinar cuáles son las condiciones en que la población vive y desarrolla su actividad. Con esto quiero ejemplificar lo que he intentado decir en repetidas oportunidades a lo largo de esta charla: no es en la relación del médico con el enfermo donde se resuelven los problemas de salud, sino en la consideración de los problemas del pueblo y esto se hace a través del trabajo en equipo, a través de mirar realmente cuál es el país donde estamos viviendo, cuáles son sus condiciones, cuáles son sus características y cómo nosotros nos metemos dentro de ese problema.

A los compañeros:

Compañeros estudiantes: esto es todo lo que tengo para decirles hoy. Quiero volver a expresarles mi más cordial bienvenida y volver a exigirles un esfuerzo que va a ser considerable, que va a ser duro porque significa no solo que van a tener que estudiar mucho, sino que ustedes personalmente van a tener que cambiar para que podamos seguir adelante y construir el país que queremos, en un futuro que no va a ser muy lejano porque ahora cuenta con el apoyo de nuestro líder, el General Perón.

Con estas palabras reitero mi bienvenida y exijo esfuerzo, trabajo y crítica hacia la gestión que nosotros, o los que nos sigan, desarrollemos desde el gobierno de esta casa.

Nada más.
